# Health needs of migrant female head porters in Ghana: evidence from the Greater Accra and Greater Kumasi Metropolitan areas

**DOI:** 10.1186/s12939-023-01947-x

**Published:** 2023-08-08

**Authors:** Rhanda Kyerewaa Opuni, Dina Adei, Anthony Acquah Mensah, Ronald Adamtey, Williams Agyemang-Duah

**Affiliations:** 1 Department of Planning, Kwame Nkrumah University of Science and Technology, Kumasi, Ghana; 2 Department of Geography and Planning, Queen’s University, Kingston, ON Canada

**Keywords:** Migrant female head porters, Health needs, Mental health, Sexual health, Ante-natal care, Post-natal care, Health policy, Ghana

## Abstract

**Background:**

In low-and middle-income countries, migrants are confronted with health needs which affect the promotion of their well-being and healthy lives. However, not much is known about the health needs of migrant female head porters (Kayayei) in Ghana. This study assesses the health needs of migrant female head porters in the Greater Kumasi Metropolitan Area (GKMA) and Greater Accra Metropolitan Area (GAMA).

**Methods:**

The study adopted a convergent mixed methods design where both qualitative and quantitative data were used. A representative sample size of 470 migrant female head porters was used for the study.

**Results:**

The study revealed that ante-natal care, post-natal care, treatment of malaria, treatment of diarrhoea diseases, mental health, sexual health, and cervical cancer were health needs of migrant female head porters. The findings showed that participants from the GAMA significantly have greater cervical cancer needs (71.6% vrs 67.1%, p = 0.001) compared to those from the GKMA. Kayeyei from the GKMA significantly have greater mental health needs than those from the GAMA (84.6% vrs 79.2%, p = 0.031). Also, Kayeyei from the GKMA significantly have higher attendance of post-natal care compared to those from the GAMA (99.4% vrs 96.2%, p = 0.013).

**Conclusion:**

The findings underscore differential health needs across geographical localities. Based on the findings of the study, specific health needs such as ante-natal care and post-natal care should be included in any health programmes and policies that aim at addressing health needs of migrant female head porters in the two metropolitan areas of Ghana.

## Introduction

Global migration statistics for the last three decades indicate an increase in the proportion of female migrants [1, 2]. The trend is nearly the same across Sub-Saharan Africa, and a major feature is that migrants migrate to urban areas [1, 2]. The literature reveals an association between migration and health [3], and is seen as a priority in public health, as well as a determinant of health [4–6]. Migration affects males and females differently due to gender issues; females are largely marginalised compared to their male counterparts [7, 8]. Lattof et al. [3] indicate that migrant female head porters are faced with challenging health issues due to limited income.

According to World Health Organization (WHO) [9], female migrants experience specific health issues such as the health and nutrition of newborns, maternal health, and sexual and reproductive health. Carballo & Mboup [10] reveal that female migrants have poor access to gynecological care and delayed healthcare delivery because they have difficulty communicating with healthcare practitioners. Female migrants experience high maternal deaths and poor pre-and post-natal care due to low socioeconomic status [11]. Anxiety and depression, for example, have been shown to be health issues experienced by female migrant populations [12, 13]. Chandra [14] argues that female migrants have higher rates of psychiatric disorders and stress among migrant population. These specific health-related issues constitute health needs faced by migrant populations.

Health needs form part of health-related issues faced by the individual [15]. According to Cavanagh & Chadwick [15], health needs refer to the perceptions of how people feel and how they express their needs. In this paper, health needs of migrant female head porters is defined as health-related issues faced by migrant female head porters and the perceptions of how they feel. Hoffer & Edgar [16], suggest that the symptoms approach needs to be used to measure the health needs of migrant women. This approach can be used by posing questions to female migrants to know their health status and which medical assistance can be given to them. Consistent with Hoffer & Edgar [16], health needs of kayayei were measured by the symptoms approach whereby they were asked questions about their health status. The health-related issues of migrant female head porters that were considered in this study were based on variables considered in WHO [17] and City and Hackney [18].

In Ghana, the trajectory of migration has been the movement of people from the northern to the southern part of the country. The Ghana Statistical Service [19] defines a migrant as a person who is living outside his/her place of birth for more than six months. Females migrate at a higher rate than males from rural to urban areas in the country [19, 20]. The majority of female head porters migrate from the Northern Region increasing their net losses [19]. The situation is attributed to the development path of Ghana. The northern part of the country is associated with low level of development, with minimal work opportunities and deprivation of basic amenities such as potable drinking water and healthcare facilities, in contrast to the southern part of the country, which has higher level of development and a large informal working sector [19]. In this study, female head porters who have moved from the northern parts of Ghana to Greater Kumasi and Greater Accra Metropolitan Areas were the study population. They are known locally as “Kayayei", and for the purposes of this study the term “Kayayei” will be used interchangeably with migrant female head porters [20].

Despite the association between migration and health, the literature on health needs of female migrants in Ghana are scant [although they are a vulnerable population] [21]. The literature on migrant female head porters has centred on barriers to healthcare access and utilisation [21–23], accessibility and utilisation of maternal health services [24], enrolment in the National Health Insurance Scheme [NHIS] [25, 23], health-seeking behaviour [26] and constraints to family planning uptake [27]. Thus, not much is known about the health needs of the migrant female population in Ghana. This study seeks to contribute to the literature on health needs of migrant female head porters in the two largest metropolitan areas in Ghana. The study’s rationale is as follows; first, unearthing health needs among women is important from a societal perspective in order to reduce health inequality and promote well-being; second, understanding the health needs of migrant female head porters could inform the formulation of health policies or programmes that addresses health-related issues faced by migrant female head porters. This could help prioritise the allocation of resources towards improvement in migrant female health needs and overall health outcome; lastly, the implementation of the results from this research may be important to contributing to the realisation of the United Nation’s health-related Sustainable Development Goals.

## Methodology

### Setting

The geographical scope of this study was the Greater Accra Metropolitan Area (GAMA) and the Greater Kumasi Metropolitan Area (GKMA). The two areas were chosen based on Ghana Statistical Service [19] census data, which reveal that these are the most urbanised areas, with most job opportunities and the lowest poverty levels in the country. As cities of attraction to the migrants, most female head porters migrate from the northern part of the country to these areas in search of greener pastures [28]. Data collection took place from January to March 2021.

### Data and sample

The convergent mixed methods design was adopted for this study. Cochran’s formula for estimating an unknown population’s sample size was used due to the unavailability of statistics on the population size of migrant female head porters in the GAMA and GKMA. Boateng et al. [25], however, suggest that there could be as many as 50,000 migrant female head porters in Accra and Kumasi alone. A sample size of 384 was obtained. A nonresponse rate of 25% was then adjusted for the sample size resulting in a sample size of 480. However, there was a non-response of 10 (2.2%); four in the GAMA and six in GKMA. Hence, the sample size for the study was 470, with 236 from GAMA and 234 from GKMA.

Time-location sampling technique was employed in selecting migrant female head porters because they were more mobile, difficult-to-reach and adequate sampling frames for identifying them were unavailable. As the name implies, time-location sampling assumes that it is possible to reach and cover the target population at predefined locations by randomly sampling those locations at different times [29]. Migrant female head porters were selected from Central Market, Adum, Bantama Market and Onion Market in the GKMA. The Agbogbloshie Market, Kantamanto Market, Mallam Atta Market and Dome Market were also selected in the GAMA. All markets in both Metropolitan areas were listed and classified. The classification was based on the size of the land and the population of patrons to the market. The classification uncovered the major markets in both Metropolitan areas. The major markets were assigned values and randomly selected using an online random number generator. Time-location sampling technique was used to select migrant female head porters. Time-location sampling takes advantage of the fact that migrant female head porters attend a universe of venues at identifiable and specific days and times. During the reconnaissance survey, we interacted with migrant female head porters to identify venues where they were gathered. We first collected quantitative data to support the inclusion of venues in the universe of venues. This aided in the selection of venues in various markets. We then looked at the times of day when migrant female head porters could be found together. Migrant female head porters were discovered at venues between the hours of 1pm and 5pm. We counted them at the venues. The counts were completed in 30 min and multiplied by four to estimate the total number of migrant female head porters who might attend a two-hour sampling event. Duplicate visits by migrant female head porters were not counted. Furthermore, we determined the number of eligible migrant female head porters who attended the venues at 1pm and 5pm. We established membership in the head porter population to be sampled and whether migrant female head porters were eligible for the study. The inclusion criteria for the study were: (i) migrant female head porters, and (ii) between 15 and 45 years.

The face-to-face administered semi-structured interview schedule was used in collecting primary data. The questions included information on the socio-demographic characteristics of respondents such as their marital status, age, and educational attainment. It also entailed questions on the health needs such as ante-natal care, post-natal care, family planning, communicable and non-communicable diseases. A Likert scale was used to determine Kayayei experience with ante-natal care which includes very good, good, fair, poor and very poor. Averagely, the face-to-face administered semi-structured interview schedule lasted between 35 and 50 min.

The semi-structured interview schedules were pre-tested with 30 migrant female head porters in the Kaneshie Market in Accra [30]. The Kaneshie Market in Accra was selected for pre-test due to the small number of migrant female head porters identified during the reconnaissance survey and the similarity of characteristics as the target population for this study. Modifications were made to the semi-structured interview schedule before the actual data collection exercise. The semi-structured interview schedules were translated from English to Hausa, Dagaare and Dagbani (local dialect understood by the respondents) following the World Health Organization guidelines for instruments assessments [31].

In this study, health needs of Kayayei were our outcome variable. Health needs of Kayayei was determined using variables such as ante-natal care, post-natal care, family planning, non-communicable diseases [high blood pressure, mental health, sexual health, cervical screening and diabetes] and communicable diseases [malaria, hepatitis B, hepatitis C, tuberculosis, Covid-19 and treatment of diarrhoea disease]. Premised on all of the above, the perception of participants on how they felt with health-related issues was assessed.

### Data analysis

Statistical analyses were performed using the IBM Statistical Package for the Social Sciences (SPSS) version 21. The descriptive (percentages and frequencies) and inferential statistics (Fisher Exact test and Chi-square tests) embedded in IBM Statistical Package for the Social Sciences (SPSS) v21 were used to analyse the quantitative data. Fisher’s exact test results were produced for 2 × 2 contingency table with low cell count [expected frequencies less than 5]. Chi-square tests results were produced for variables with more than 2 categories and expected frequencies greater than 5. The level of significance thresholds for all analyses of the association were pegged at 0.05. Results were presented in the form of tables. Qualitative data were organised after processing and screening. We adapted the steps of thematic analysis, namely familiarisation with the data, generating initial codes, searching for themes that align with the study variables, and reporting. By integrating both quantitative and qualitative data, an in-depth and reliable understanding was obtained regarding the health needs of female migrant head porters. The integration of data was achieved through the weaving approach which involved the use of narratives when presenting the results. As such, the results from the quantitative survey and the qualitative interviews were organised thematically (namely ante-natal care, post-natal care, family planning, non-communicable diseases, and communicable diseases) and classified according to the study variables.

#### Ethical approval

for this research was granted by Committee on Human Research Publication and Ethics (CHRPE), School of Medical Sciences, Kwame Nkrumah University of Science and Technology and Komfo Anokye Teaching Hospital, Kumasi (Ref: CHRPE/AP/307/21). Informed consent was sought from respondents before the study commenced. With regard to the minors’, informed consent was obtained from their parents or legal guardians which was approved by the institutional review board.

## Results and discussion

This section presents and discusses the results on health needs of Kayayei. Given that the socio-demographic characteristics of migrant female head porters can have a significant impact on their health needs, the section begins with findings on socio-demographic characteristics of migrant female head porters.

### Socio-demographic characteristics of migrant female head porters

Table 1 depicts that the majority of Kayayei (72.4%) were between the ages of 18–29 years. About 55% of Kayayei had no formal education, with majority (83%) unable to read and write. Approximately 57% of Kayayei were married and 58.1% earned between GH¢ 301–600 monthly (US$ 39.6–79.0, exchange rate as at April 2022 was GH¢7.60 = US$1.00). Kayayei constitute a youthful and economically active population because more than half of participants (72.4%) fell within the age group of 18 to 29 years. This confirms a similar study by Opare [32] that Kayayei constitute a youthful and economically active population. The study revealed that young females tend to migrate more than elderly ones, which is consistent with the findings of Lattof et al. [21]. Thus, head porterage is predominantly confined to young females [20].

**Table 1:** Socio-demographic Characteristics of Kayayei

Variables	Location				Total	
	Greater Kumasi Metropolitan Area		Greater Accra Metropolitan Area			
	No.	%	No.	%	No.	%
**Age in years**						
Below 18	32	13.7	58	24.6	90	19.1
18–29	196	83.7	144	61.0	340	72.4
30–39	6	2.6	34	14.4	40	8.5
**Sub-total**	**234**	**100.0**	**236**	**100.0**	**470**	**100.0**
**Educational Attainment**						
No formal education	146	62.4	111	47.0	257	54.7
Primary	30	12.8	37	15.7	67	14.3
Junior High school	58	24.8	88	37.3	146	31.0
**Sub-total**	**234**	**100.0**	**236**	**100.0**	**470**	**100.0**
**Marital Status**						
Married	138	59.0	129	54.7	267	56.8
Single	96	41.0	107	45.3	203	43.2
**Sub-total**	**234**	**100.0**	**236**	**100.0**	**470**	**100.0**
**Literacy (Able to read and write)**						
Yes	17	7.3	63	26.7	80	17.0
No	217	92.7	173	73.3	390	83.0
**Sub-total**	**234**	**100.0**	**236**	**100.0**	**470**	**100.0**
**Income**						
GH¢ 1–300	1	0.4	38	16.1	39	8.3
GH¢ 301–600	135	57.7	138	58.5	273	58.1
GH¢ 601–800	98	41.9	60	25.4	158	33.6
**Sub-total**	**234**	**100.0**	**236**	**100.0**	**470**	**100.0**
**NHIS**						
Yes	106	45.3	95	40.3	201	42.8
No	128	54.7	141	59.7	269	57.2
**Sub-total**	**234**	**100.0**	**236**	**100.0**	**470**	**100.0**

### Health needs of migrant female head porters

#### Ante-natal care

Ante-natal care is a means through which healthcare practitioners are able to identify high risk pregnancies and also educate women to help them have healthy and safe delivery [33]. Table 2 shows that 71.5% of the Kayayei have been pregnant before; with 75.6% and 67.4% of them from GKMA and GAMA, respectively. All Kayayei in GKMA who have been pregnant before attended ante-natal care while 96.2% in GAMA attended ante-natal care. All Kayayei (100%) included ante-natal care as their health need (see Table 2). A statistically significant difference exists between the location of Kayayei, being pregnant before and attending their ante-natal care appointments (*p*-value = 0.041, *p*-value = 0.003).

**Table 2:** Data on Kayayei Ante-Natal Care

Variables	Location						*P*-value
	Greater Kumasi Metropolitan Area		Greater Accra Metropolitan Area		Total		
	No.	%	No.	%	No.	%	
**Being Pregnant Before**							
Yes	177	75.6	159	67.4	336	71.5	0.041
No	57	24.4	77	32.6	134	28.5	
**Sub-total**	**234**	**100.0**	**236**	**100.0**	**470**	**100.0**	
**Attend Ante-Natal Care**							
Yes	177	100	153	96.2	330	98.2	0.003
No	0	0.0	6	3.8	6	1.8	
**Sub-total**	**177**	**100.0**	**159**	**100.0**	**336**	**100.0**	
**Attending all Ante-Natal care Appointments**							
Yes	14	7.9	20	13.0	34	10.3	0.008
No	163	92.1	133	87.0	296	89.7	
**Sub-total**	**177**	**100.0**	**153**	**100.0**	**330**	**100.0**	
**Period of Starting Ante-Natal Care**							
First Trimester	61	34.5	72	47.1	133	40.3	0.003
s Trimester	116	65.6	80	52.3	196	59.4	
Third Trimester	0	0	1	0.6	1	0.3	
**Sub-total**	**177**	**100.0**	**153**	**100.0**	**330**	**100.0**	
**Experience with Ante-Natal Care**							
Very Good	173	97.7	84	54.9	257	77.9	0.000
Good	4	2.3	68	44.4	72	21.8	
Fair	0	0	1	0.7	1	0.3	
**Sub-Total**	**177**	**100.0**	**153**	**100.0**	**330**	**100.0**	
**Ante-Natal care as a Health Need**							
Yes	234	100.0	236	100.0	470	100.0	
No	0	0	0	0	0	0	
**Sub-Total**	**234**	**100.0**	**236**	**100.0**	**470**	**100.0**	

In general, migrant female head porters in the interviews expounded on the significance of ante-natal care as a crucial component for ensuring a safe delivery. They emphasised that adequate utilisation of ante-natal care could potentially prevent any fatalities that may arise from pregnancy or other complications prior to delivery. Thus, the regular attendance of ante-natal care services results in a heightened sense of security and assurance for migrant female head porters.

“*The mother and her child will be monitored and that will help the doctors find out if there are any complications associated with the pregnancy and help solve those complications.”* (Migrant female head porter A, GKMA).

A migrant female head porter concluded:

*“Attending ante-natal care is very important since it will lead to safe delivery of the baby and the mother.*” (Migrant female head porter B, GKMA).

The majority of migrant female head porters (98.2%) attended ante-natal care because of the knowledge they have from public sensitization by health officers on the benefits of ante-natal care. However, 10.3% Kayayei attended all their ante-natal care appointments, with 13.0% from GAMA and 7.9% from GKMA (see Table 2). There is a statistically significant association between the location of Kayayei and attending all ante-natal care appointments (*p*-value = 0.008) (Table 2). Migrant female head porters explained that they were unable to attend all their ante-natal care appointments because they sometimes worked on their appointment days. Nevertheless, they revealed they tried their best to attend ante-natal care appointments because it was covered under the NHIS, thus had no toll on expenditure on healthcare. This study outcome suggests that NHIS promotes the use of healthcare. The finding corroborates that of previous studies on the association of NHIS and the utilization of healthcare services and the adoption of good health-seeking behaviour [34, 35].

Approximately 59% of the Kayayei started their ante-natal care in their second trimester (Table 2). However, no Kayayei in GKMA attended ante-natal care in their third trimester compared to one Kayayo [singular form of Kayayei] who attended ante-natal in her third trimester in GAMA. WHO [33] recommends that pregnant women start ante-natal care appointments in the 12th week of pregnancy, which falls within their first trimester [known as early ante-natal care]. However, approximately 59% of migrant female head porters did not start ante-natal care in the early ante-natal care period [first trimester], hence falling short of the WHO recommendation [30]. This study’s outcome suggests delayed first ante-natal care visit. This finding is congruent with a previous study that indicated delayed first ante-natal visits [36]. Women with no formal education, with unintended pregnancy and perceived that the right initiation time of the first antenatal care visit is beyond 16 weeks of gestation were likely to delay on their first antenatal care visit [36, 37]. All migrant female head porters attended at least 4 of their ante-natal care appointments. The finding is in conformity to Lincento et al. [38], who report that the success story of ante-natal care in Africa is that about 69% of women have at least one ante-natal care contact with their healthcare practitioners.

The study revealed that 77.9%, 21.8% and 0.3% of migrant female head porters rated their experience with ante-natal care as very good, good and fair, respectively (see Table 2). None in both metropolitan areas rated their experience with ante-natal care as poor and very poor. However, Kayayei in GKMA gave higher ratings (97.7%) for their experience with rating it as very good compared to 54.9% rating of very good in GAMA. No Kayayie in GKMA rated their experience as fair while one Kayayo in GAMA rated her experience as fair. There is a statistically significant association between the location of Kayayei and their experience with ante-natal care.

#### Post-natal care

Post-natal care is important because it ensures that care is given to the new mother and her new born child [33]. In this study, therefore, an attempt was made to find out if migrant female head porters received post-natal care after pregnancy. Approximately, 98% of Kayayei attended their post-natal care appointments; with 99.4% and 96.2% in GKMA and GAMA, respectively (see Table 3). There was a statistically significant association between the location of Kayayei and attending their post-natal care appointments (*p*-value = 0.013) (Table 3). Attending post-natal care appointments is essential because it helps doctors keep an eye on how the nursing mother is healing after childbirth, check if the baby is growing healthily and further start with the baby’s immunisation [33]. The majority of migrant female head porters (86.3%) attended all their post-natal care appointments and they all indicated post-natal care as their health need (Table 3). This finding is in line with WHO [33] recommendation that nursing mothers must attend at least 4 of their post-natal care appointments. However, more Kayayei in GAMA (86.9%) attended all their post-natal care appointments in comparison to 85.8% of Kayayei in GKMA (Table 3). There is a statistically significant association between the location of Kayayei and attending all their post-natal care appointments (*p*-value = 0.033) (Table 3).

**Table 3:** Data on Kayayei Post-Natal Care

	Location							P-value
Variables	Greater Kumasi Metropolitan Area		Greater Accra Metropolitan Area		Total			
	No.	%	No.	%	No.		%	
**Attend Post-Natal Care**								
Yes	176	99.4	153	96.2	329		97.9	
No	1	0.6	6	3.8	7		2.1	0.013
**Sub-total**	**177**	**100.0**	**159**	**100.0**	**336**		**100.0**	
**Attending All Post-Natal care Appointments**								
Yes	151	85.8	133	86.9	284		86.3	
No	25	14.2	20	13.1	45		13.7	0.033
**Sub-total**	**176**	**100.0**	**153**	**100.0**	**329**		**100.0**	
**Period of Starting Post-Natal Care**								
First month after delivery	176	100	144	94.1	320		97.3	
s to fourth month Trimester	0	0	9	5.9	9		2.7	0.000
**Sub-total**	**176**	**100.0**	**153**	**100.0**	**329**		**100.0**	
**Experience with Post-Natal Care**								
Very Good	173	98.3	86	56.2	259		78.7	
Good	3	1.7	66	43.1	69		21.0	0.000
Fair	0	0	1	0.7	1		0.3	
**Sub-total**	**176**	**100.0**	**153**	**100.0**	**329**		**100.0**	
**Post-Natal Care as a Health Need**								
Yes	234	100.0	236	100.0	470		100.0	
No	0	0	0	0	0		0	
**Sub-Total**	**234**	**100.0**	**236**	**100.0**	**470**		**100.0**	

A general picture that emerged from the interviews is that the attendance of post-natal care appointments by migrant female head porters was driven by their knowledge of the benefits that could be obtained from post-natal care. Migrant female head porters said they saw the added value of post-natal care for themselves and their child. They found post-natal care necessary and useful for the healthy development of the child. For them it was evident that the post-natal period was a critical stage after childbirth, during which the baby is immunised against life-threatening childhood diseases such as measles, pertussis, diphtheria, tetanus, tuberculosis and poliomyelitis, and the mother is educated on how to take good care of the child.

*“The mothers are educated on how to take care of the baby and the baby is also immunized against killer diseases which will enable the child grow healthy and strong”* (Migrant female head porter A in GAMA).

About 97% of Kayayei started their post-natal care first month after delivery (see Table 3). Further analysis revealed that all Kayayei in GKMA commenced their post-natal care appointments in the first month after delivery compared to 94.1% in GAMA. There is a statistically significant association between the location of Kayayei and period of starting their post-natal care appointments (*p*-value = 0.000) (Table 3). WHO [33] recommends that mothers start their post-natal care appointments early within the first 24 hours after delivery and subsequent care within the first month after delivery. The findings indicate that migrant female head porters followed WHO [33] recommendation in starting their post-natal care appointments to ensure care is given to the mother and her new born child to prevent maternal and child death. It is, therefore, not surprising when all Kayayei (100%) indicated post-natal care as their health need. This study found that Kayayei do not incur costs during their post-natal care appointments because it is covered under the NHIS. This served as a motivation for Kayayei to attend their post-natal care appointments. All migrant female head porters who had ever been pregnant before revealed that the NHIS covers the costs of their post-natal care appointments. This finding resonates with the argument that migrant female head porters are able to attend their post-natal care because it is free for all under the NHIS [39].

Approximately 98% and 2% of Kayayei in GKMA rated their post-natal care experience as very good and good, respectively compared to 56.2% and 43.1% of Kayayei in GAMA. The results indicate that there is a statistically significant association between the location of Kayayei and experience with post-natal care appointments. None of the Kayayei rated their experience with post-natal care as poor and very poor. The implication is that Kayayei do not encounter major barriers that affect their experience with post-natal care appointments.

### Family planning

Unlike female non-migrants who indicate family planning as a health need [40], approximately 82% of Kayayei in this study did not identify it as a health need (Table 4). In comparison, 85% of Kayayei in GKMA did not include family planning as their health need compared to 78% Kayayei in GAMA (see Table 4). This implies that family planning was not a major health need among migrant female head porters in both cities. Approximately 86% of migrant female head porters in the study area did not practice family planning; 87.2% and 84.3% in GKMA and GAMA, respectively. The results revealed that there was a statistically insignificant association between the location of Kayayei and the practice of family planning. The finding supports what has been noted in the literature that migrant female head porters do not practice family planning in cities in Ghana [40].

**Table 4:** Data on Kayayei family Planning Practices

	Location						P-value
	Greater Kumasi Metropolitan Area		Greater Accra Metropolitan Area		Total		
	No.	%	No.	%	No.	%	
**Family Planning as a Health Need**							
Yes	35	15.0	52	22.0	87	18.5	
No	199	85.0	184	78.0	383	81.5	0.001
**Sub-total**	**234**	**100.0**	**236**	**100.0**	**470**	**100.0**	
**Practice of Family Planning**							
Yes	30	12.8	37	15.7	67	14.3	0.430
No	204	87.2	199	84.3	403	85.7	
**Sub-total**	**234**	**100.0**	**236**	**100.0**	**470**	**100.0**	

Regarding the practice of family planning, cultural, religious and social factors play a significant role in shaping the perception of family planning of migrant female head porters. Even though acknowledging the substantial advantages of family planning, migrant female head porters regarded their religion, culture, and societal norms as impediments to its practice. For instance, these head porters expressed their apprehension towards being ostracized or demeaned for having a limited number of children. Furthermore, some migrant head porters used the Quaran and the Bible to rationalize their decision to abstain from family planning.

*“It is stated in the Quaran that we should multiply and fill the earth, why then must I do contrary to that?”* (Migrant female head porter B in GAMA)

*“I do not want a situation whereby people will insult me using the number of kids I have by telling me that is why I cannot have many children. So, I will not do it so I can have many children”* (Migrant female head porter C in GAMA).

The Ghanaian society is shaped by culture and religious affiliations. People tend to make lifetime decisions based on these cultures and religious beliefs which have both positive and negative effects on their lives. For instance, as a result of cultural and religious beliefs, Kayayei in this study revealed that they stayed faithful to their partners because their religion forbids them from doing otherwise. However, this same religion speaks against the use of family planning, thus they also do not practice family planning methods, notwithstanding the positive benefits derived from practicing family planning such as spacing of children and having more productive hours to work due to fewer number of children [33]. Migrant female head porters looked past these benefits because of their cultural and religious beliefs, which has implication on their finances and well-being.

### Non-communicable diseases

Non-communicable diseases keep increasing in Ghana [41]. Non-communicable diseases result from lifestyle, foods consumed and also from genetics [41]. Non-communicable diseases discussed in this study include high blood pressure, mental health, sexual health, cervical cancer and diabetes. Approximately 25% of Kayayei had knowledge of their blood pressure level, with 98.3% checking their blood pressure level at the hospital. All respondents (100%) indicated that health professionals had informed them that they have a normal blood pressure level. There is a statistically significant association between the location of Kayayei and knowledge of their blood pressure level (*p*-value = 0.000) (see Table 5). The majority of Kayayei (75.1%) did not know their blood pressure level and this could be attributed to Kayayei not going for medical checkup due to the long waiting time they have to spend at the hospital. Yiran [24] confirms this by indicating that Kayayei do not have knowledge of their blood pressure level. Yiran [24] indicates that the long hours of waiting at health facilities prevents Kayayei from seeking for healthcare services. As such they were unaware of their blood pressure level. According to MedlinePlus [42] knowing one’s blood pressure level is essential to living a healthy lifestyle. Kayayei should be encouraged to check their blood pressure level because of the nature of their work [carrying heavy loads]. Petersen et al. [43] assert that occupational heavy lifting is associated with acute cardiovascular strain excessively raising blood pressure. Therefore, Kayayei’s knowing their status will decrease their risk of serious health problems, including heart attack and stroke [41, 44].

**Table 5:** Data on Kayayei’ Non-Communicable Diseases

	Location							P-value
Variables	Greater Kumasi Metropolitan Area		Greater Accra Metropolitan Area		Total			
	No.	%	No.	%	No.		%	
**Knowledge of Blood Pressure Level**								
Yes	82	35.0	35	14.8	117		24.9	
No	152	65.0	201	85.2	353		75.1	0.000
**Sub-total**	**234**	**100.0**	**236**	**100.0**	**470**		**100.0**	
**Place of Checking Blood Pressure Level**								
Hospital	82	100.0	33	94.3	115		98.3	
Pharmacy	0	0.0	2	5.7	2		1.7	0.001
**Sub-total**	**82**	**100.0**	**35**	**100.0**	**117**		**100.0**	
**Blood Pressure Level**								
Normal	82	100.0	35	100.0	117		100.0	
**Sub-total**	**82**	**100.0**	**35**	**100.0**	**117**		**100.0**	
**Mental Health Need**								
Yes	198	84.6	187	79.2	385		81.9	
No	36	15.4	49	20.8	85		18.1	0.031
**Sub-Total**	**234**	**100.0**	**236**	**100.0**	**470**		**100.0**	
**Sexual Health Need**								
Yes	149	63.7	139	58.9	288		61.3	
No	85	36.3	97	41.1	182		38.7	0.508
**Sub-total**	**234**	**100.0**	**236**	**100.0**	**470**		**100.0**	
**Seeking Sexual Health Services**								
Yes	151	64.5	149	63.1	300		63.8	
No	83	35.5	87	36.9	170		36.2	0.601
**Sub-Total**	**234**	**100.0**	**236**	**100.0**	**470**		**100.0**	
**Cervical Cancer Health Need**								
Yes	157	67.1	169	71.6	326		69.4	
No	77	32.9	67	28.4	144		30.6	0.000
**Sub-total**	**234**	**100.0**	**236**	**100.0**	**470**		**100.0**	
**Diabetes as a Health Need**								
Yes	0	0.0	2	0.8	2		0.4	
No	234	100.0	234	99.2	468		99.6	0.002
**Sub-total**	**234**	**100.0**	**236**	**100.0**	**470**		**100.0**	
**Having Diabetes**								
Yes	0	0	0	0	0		0	
No	234	100.0	236	100.0	470		470	
**Sub-total**	**234**	**100.0**	**236**	**100.0**	**470**		**100.0**	

Mental health, in this study, refers to an individual’s condition regarding his or her emotional and psychological well-being [45, 46]. Women are more likely to have mental health problems compared to men [45]. Hence, it was unsurprising that migrant female head porters indicated mental health as their health need. In the survey, about 82% Kayayei indicated mental health as their health need, with 84.6% from GKMA and 79.2% from GAMA (Table 5). There is a statistically significant association between the location of Kayayei and mental health need (*p*-value = 0.031) (see Table 5). All Kayayei indicated they have been depressed or sad in the last four weeks preceding the study.

Like the survey results, study participants mentioned mental health concerns, including depression and having suicidal tendencies. They stated that the high cost of living in the cities often engenders depression, as individuals in such environments struggle to meet their daily sustenance needs. Furthermore, the study participants noted that this struggle might lead individuals to return to their hometown (rural areas), where poverty is even more pronounced.

*“My friend who is also a Kayayei got pregnant, and she was scared about what her parents will do to her. She stopped eating and working. One morning when we all woke up, she was still asleep and when I went to wake her up, she did not wake up. We rushed her to the hospital and we were told she overdosed on some medicine. My friend killed herself because she was going through a lot.”* (Migrant female head porter D in GAMA).

The findings of this study are consistent with European Network of Migrant Women [47] study which revealed that female migrants have mental health issues due to them being sad and depressed. People who are depressed tend to have suicidal thoughts [45].

Sexual health needs refer to the state of emotional, physical, social and mental well-being of a person in relation to sexuality [33]. Approximately 61% Kayayei indicated sexual health as their health need; 63.7% and 58.9% from GKMA and GAMA, respectively (Table 5). However, there was a statistically insignificant association between the location of Kayayei and sexual health need (*p*-value = 0.508) (Table 5). Including sexual health as a health need shows that Kayayei want their sexual health needs to be adequately met as such they all indicated sexual health as their health need with 63.8% haven sought for sexual health services from a health facility (Table 5).

Migrant female head porters were more positive about their sexual health needs. They expressed their conviction that sexual health is indispensable and efficacious in the prevention of sexually transmitted infections including syphilis, herpes and HIV andAIDS, as well as in ensuring the well-being of their reproductive system, thereby facilitating successful childbearing.

*“I am scared of getting HIV andAIDS, so I want to know what to do so that I will not get this disease.”* (Migrant female head porter B).

*“I want to give birth to plenty children, so I want to make sure that everything within my reproductive system is good.”* (Migrant female head porter C).

There is a close linkage between sexual health and reproductive health [7]. Kayayei sought sexual health services on pregnancy and sexually transmitted diseases. This finding aligns with Loganathan et al. [48] that indicates that female migrants seek sexual health services pertaining to pregnancy and sexually transmitted diseases. This shows the importance they give to their sexual health since they want to have in-depth knowledge about it. Sexual health is essential because it gives an individual the opportunity to take charge of their reproductive health and emotional well-being which surrounds their intimate relationships [49].

The study revealed that migrant female head porters had cervical cancer health needs. The World Health Organization [33] defines cervical screening as the screening of a woman’s cervix to identify any changes in the cervix’s cell that could lead to cancer. About 69% of Kayayei indicated cervical screening as a health need; with 71.6% and 67.1% in GAMA and GKMA, respectively (see Table 5). There is a statistically significant association between the location of Kayayei and cervical cancer as a health need (*p*-value = 0.000) (see Table 5).

Expounding upon cervical health needs, migrant female head porters conveyed that undergoing cervical health screening would enable the detection of precancerous cells which bear the potential to escalate into cervical cancer. Furthermore, they affirmed that such screening measures would help physicians in determining whether any complications exist within the uterine environment which could pose a threat to the process of childbirth.

*“I do not want anything to prevent me from giving birth. Going for cervical screening will ensure that the doctors are able to see if there is something wrong with my womb and give me medications to heal me.”* (Migrant female head porter D).

Although majority of Kayayei revealed cervical cancer as their health need, programmes on cervical cancer in developing countries including Ghana have not yet been implemented and the cost of cervical screening is not free [50, 51]. This is, however, contrary to Jackowska et al. [52] finding which state that cervical screening is free for migrant women in London and they get periodic reminders to go for their check-ups. This study’s outcome calls attention on policy makers to formulate policies on cervical cancer to ensure Kayayei cervical health needs are adequately met. This will ensure that female migrant head porters are healthy and free from cervical cancer and subsequently be of sound mind to work efficiently and contribute their quota to the development of their country [7].

Diabetes refers to high levels of blood sugar in an individual which could result in damage to the heart, eyes, blood vessels, nerves and kidneys [7]. Only two Kayayei indicated diabetes as their health need; with no Kayayo in GKMA (Table 5). There is a statistically significant association between location of Kayayei and diabetes as a healthcare need (*p*-value = 0.002) (see Table 5). No Kayayei had diabetes (see Table 5), which indicates that diabetes was not considered a health need.

Regarding diabetes as a health need, migrant female head porters exhibited a negative attitude towards it. The rationale presented by them was that as they did not personally suffer from diabetes, they did not perceive the necessity to partake in routine health screenings at health facilities in order to determine their status. Furthermore, they did not feel compelled to seek counsel from physicians regarding preventive measures.

*“I do not have diabetes, so there is no need to include this as my health need.”* (Migrant female head porter F in GAMA).

Nevertheless, Kayayei should be encouraged to live and maintain a healthy lifestyle to reduce their risk of disease as they age. This can be done through routine sensitisation programmes targeted at migrant female head porters. Our results are contrary to findings from Shah et al. [53] which indicate that migrant women in United Arab Emirates have a higher prevalence of diabetes compared to the indigenes. The difference in variations is as a result of the unit of analysis as well as the study setting. This study used Kayayei as the unit of analysis whereas Shah et al. [53] used all migrant women living in United Arab Emirates.

### Communicable diseases

Communicable diseases considered in this study were malaria, hepatitis B, hepatitis C, tuberculosis, Covid-19 and treatment of diarrhoea disease. The study found that all Kayayei (100%) in the two cities had contracted malaria 6 months prior to the data collection exercise (see Table 6). The findings support Ahlvin [54] study which revealed that female migrants frequently contract malaria in *Agbobloshie*. This is attributed to the fact that Kayayei live in a deplorable environment where sanitation conditions are poor. According to Makorni [55], Kayayei live in bad conditions by sleeping on the streets and in front of shops and for those with housing accommodations, these houses are in bad conditions. Migrant female head porters sparingly sleep under mosquito nets as they sleep in vulnerable areas such as sleeping on the streets, in front of shops and in wooden structures popularly known as “Kuos” [21].

**Table 6:** Data on Kayayei Communicable Diseases

	Location					
Variables	Greater Kumasi Metropolitan Area		Greater Accra Metropolitan Area		Total	
	No.	%	No.	%	No.	%
*Malaria						
Yes	234	100.0	236	100.0	470	100.0
No	0	0.0	0	0.0	0	0.0
**Sub-total**	**234**	**100.0**	**236**	**100.0**	**470**	**100.0**
*Hepatitis B						
Yes	0	0.0	0	0.0	0	0.0
No	234	100.0	236	100.0	470	100.0
**Sub-total**	**234**	**100.0**	**236**	**100.0**	**470**	**100.0**
*Hepatitis C						
Yes	0	0.0	0	0.0	0	0.0
No	234	100.0	236	100.0	470	100.0
**Sub-total**	**234**	**100.0**	**236**	**100.0**	**470**	**100.0**
*Tuberculosis						
Yes	0	0.0	0	0.0	0	0.0
No	234	100.0	236	100.0	470	100.0
**Sub-total**	**234**	**100.0**	**236**	**100.0**	**470**	**100.0**
*Covid-19						
Yes	0	0.0	0	0.0	0	0.0
No	234	100.0	236	100.0	470	100.0
**Sub-total**	**234**	**100.0**	**236**	**100.0**	**470**	**100.0**
**Treatment Diarrhoea Disease**						
Yes	228	97.4	200	84.7	428	91.1
No	6	2.6	36	15.3	42	8.9
**Sub-Total**	**234**	**100.0**	**236**	**100.0**	**470**	**100.0**

Approximately 91% of Kayayei indicated treatment of diarrhoea disease as a health need with 97.4% and 84.7% from GKMA and GAMA, respectively (Table 6). This goes further to show that diarrhoea is a health issue facing migrant female head porter population in the two metropolitan cities. The increasing housing deficit in Ghana accompanied with high rent in cities affect the urban poor including Kayayei from accessing decent housing. The unclean surroundings in which Kayayei reside are prone to a high population of flies, leading to contamination of their food and ultimately cause them to contract diarrhoea diseases. The incidence of diarrhoea disease among Kayayei may have a deleterious effect on their productivity, as their ability work for extended periods of time may be significantly reduced, resulting in a decline in their overall income levels. None of the respondents had contracted Hepatitis B, Hepatitis C, Covid-19 or tuberculosis before, thus did not indicate it as a health need.

### Strengths and limitations

To the best of our knowledge, this is one of the first studies that explore health needs of migrant female head porters in Ghana. Despite the contributions of this study, some limitations are notable. Our findings may be exposed to a recall bias as the data was self-reported. The study did not use a standardised tool in measuring health needs of Kayayei. Also, the researchers used six months estimation to measure health needs hence there could be over-reporting or under-reporting on the part of participants. We recommend that future studies should focus on longitudinal data to measure health needs of migrant female head porters.

### Implications for policy, practice and research

Based on the findings of the study, policy, practice, and research implications of the study have been highlighted. Given the findings of the study, in terms of the policy, we propose that the formulation of a health policy that focuses on the health needs of migrant females should include needs such as ante-natal care, post-natal care, mental health, cervical cancer, sexual health, treatment of diarrhoea and malaria. This is because these are the significant health needs of Kayayei hence, it is important to capture their health needs in any health policy.

With regard to practice, the District Health Directorates and the Assemblies should educate, sensitise and organise health awareness campaigns on healthcare. The education, sensitisation and awareness should primarily focus on the health needs of women. Based on the findings of this study, the health needs should target Kayayei in GKMA and GAMA. Again, our findings indicate that the majority of Kayayei do not practice family planning and also do not start their ante-natal care early in the first trimester as recommended by [30]. Thus, as part of the health awareness campaigns and sensitisation, the District Health Directorates and the Assemblies should educate Kayayei on the benefits of practicing family planning methods and starting their ante-natal care in their first trimester. To the best of our knowledge, this will ensure that Kayayei have knowledge about the benefits of practicing family planning and subsequently have control over the number and spacing of their children and also have an early detection if there is something wrong with their unborn child. This education should be done at least once in a month in the market centers because Kayayei are found there.

Lastly, concerning the research implications, future research should focus on predictors of health needs of Kayayei. These predictors can be variables such as socio-demographic characteristics of Kayayei and their social support capital. These variables have the tendencies of influencing or predicting specific health needs of Kayayei hence it is crucial to find out what these variables are. With this, information about the predictors to their health needs will be known. Hence policy makers will factor in these variables when formulating health policies for the country.

## Conclusion

This paper examines the health needs of migrant female head porters in the GKMA and GAMA. The study found that ante-natal care, post-natal care, treatment of malaria, treatment of diarrhoea disease, mental health, sexual health and cervical cancer were health needs of migrant female head porters in GKMA and GAMA. Family planning and diabetes, however, were not the major health needs of migrant female head porters. These health needs if not addressed could negatively affect the health and well-being of migrant female head porters [who are considered as a marginalised and vulnerable population] in cities of Ghana. We argue that the formulation of polices, allocation of resources and implementation of programmes that consider the identified health needs would be useful in improving health and well-being of migrant female head porters. Attitudinal change programmes and activities such as regular orientations and sensitisation would emphasise on the importance of adopting a good health-seeking behavior for their well-being. The study has implications for health equity and health policy framework in Ghana.

## Data Availability

The datasets used and/or analyzed during the current study are available from the corresponding author on reasonable request.

## Abbreviations

GKMA: Greater Kumasi Metropolitan Area
GAMA: Greater Accra Metropolitan Area
NHIS: National Health Insurance Scheme
CHRPE: Committee on Human Research Publication and Ethics
NDPC: National Development Planning Commission
GSS: Ghana Statistical Service
